# On the Use of Mechanistic Soil–Plant Uptake Models: A Comprehensive Experimental and Numerical Analysis on the Translocation of Carbamazepine in Green Pea Plants

**DOI:** 10.1021/acs.est.0c07420

**Published:** 2021-02-15

**Authors:** Giuseppe Brunetti, Radka Kodešová, Helena Švecová, Miroslav Fér, Antonín Nikodem, Aleš Klement, Roman Grabic, Jiří Šimůnek

**Affiliations:** † Institute for Soil Physics and Rural Water Management, University of Natural Resources and Life Sciences, Vienna (BOKU), Muthgasse 18, 1180 Vienna, Austria; ‡ Faculty of Agrobiology, Food and Natural Resources, Dept. of Soil Science and Soil Protection, Czech University of Life Sciences Prague, Kamýcká 129, CZ-16500 Prague 6, Czech Republic; § Faculty of Fisheries and Protection of Waters, South Bohemian Research Center of Aquaculture and Biodiversity of Hydrocenoses, University of South Bohemia in České Budějovice, Zátiší 728/II, CZ-38925 Vodňany, Czech Republic; ∥ Department of Environmental Sciences, University of California, Riverside, California 92521, United States

## Abstract

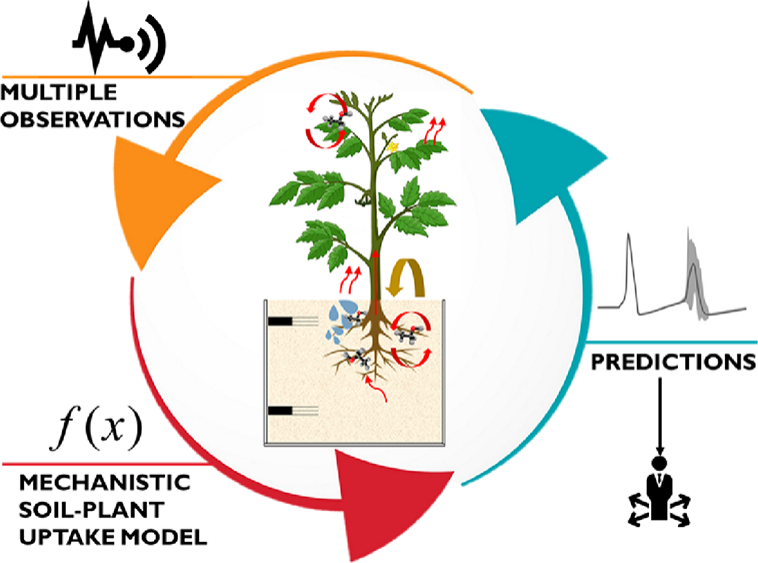

Food contamination is a major worldwide risk for human health. Dynamic plant uptake of pollutants from contaminated environments is the preferred pathway into the human and animal food chain. Mechanistic models represent a fundamental tool for risk assessment and the development of mitigation strategies. However, difficulty in obtaining comprehensive observations in the soil–plant continuum hinders their calibration, undermining their generalizability and raising doubts about their widespread applicability. To address these issues, a Bayesian probabilistic framework is used, for the first time, to calibrate and assess the predictive uncertainty of a mechanistic soil–plant model against comprehensive observations from an experiment on the translocation of carbamazepine in green pea plants. Results demonstrate that the model can reproduce the dynamics of water flow and solute reactive transport in the soil–plant domain accurately and with limited uncertainty. The role of different physicochemical processes in bioaccumulation of carbamazepine in fruits is investigated through Global Sensitivity Analysis, which shows how soil hydraulic properties and soil solute sorption regulate transpiration streams and bioavailability of carbamazepine. Overall, the analysis demonstrates the usefulness of mechanistic models and proposes a comprehensive numerical framework for their assessment and use.

## 1 Introduction

Food contamination from polluted environments represents a major risk for human health. Plants represent the most common pathway into the human and animal food chain for environmental pollutants. Chemicals are taken up by plant roots and then translocated toward the edible parts, where they are bioaccumulated^1−6^ or eventually metabolized in byproducts,^7,8^ which can be toxic.^9,10^ Soil is among the most important pollution sources for plants.^11^ It has been shown that it can be contaminated by human and veterinary pharmaceuticals,^12−14^ uptake of which is controlled by plants, chemicals, and soil properties.^15−17^

Carbamazepine (CBZ) is a widely prescribed anticonvulsant pharmaceutical and antiepileptic drug. Once administered, it is degraded by the liver in multiple metabolites, which are then excreted in both feces and urine. Due to its persistence, it is frequently encountered in water bodies.^18^ CBZ is efficiently taken up by many plants^5,7,8,19,20^ due to its chemical properties, stability, and relative mobility in the soil.^21−23^ Despite its stability in the soil, CBZ is translocated and metabolized in plants’ tissues (particularly in leaves) by plant cytochrome P450 enzymes.^5,24,25^ Root uptake and reactive transport are plant- and soil-dependent,^16,23,26^ making it difficult to generalize results. Numerical models play an important role in better understanding of physicochemical processes involved in the dynamic uptake of chemicals and risk assessment. To predict the behavior of non-ionic organic compounds in the soil–plant continuum, Brunetti et al.^27^ coupled the widely used Richards-based solver, HYDRUS-1D,^28^ with a modified version of the multicompartment dynamic plant uptake model developed by Trapp.^29^ The fully coupled soil–plant model is able to provide a comprehensive mechanistic description of transport and reaction processes in the soil–plant continuum. The model was tested for three leaf vegetables (spinach, lamb’s lettuce, and arugula). The final concentrations of CBZ and its two metabolites in only two plant tissues (i.e., roots and leaves) were measured and simulated. Despite promising results, available observations were limited to draw conclusions about the model’s applicability and generalizability.

To further test the model performance, a new, more comprehensive experiment involving the exposure of green pea plants (*Pisum sativum* L.) to CBZ was designed and carried out in a controlled environment. The green pea plant is a widespread edible vegetable, which was chosen due to its efficiency in metabolizing CBZ^23^ and its relatively fast growth and fruit formation. Extensive measurements in the soil and the plants were used in this study to calibrate the soil–plant model and assess, for the first time, its predictive uncertainty in a probabilistic Bayesian calibration framework. The calibrated model was then coupled with Global Sensitivity Analysis to identify the factors driving the accumulation of CBZ in the edible parts of the plant and shed light on the role of different physicochemical properties on the translocation of CBZ in the soil–plant continuum.

## 2 Materials and Methods

### 2.1 Case Study Description

Green pea plants (*P. sativum* L.) were planted in 24 soil columns (a height of 20 cm, a diameter of 15.4 cm) under greenhouse conditions. The soil was taken from the surface horizon (0–25 cm) of a Haplic Chernozem developed on loess (Table S1). Soil samples were first air-dried under greenhouse conditions (up to a soil–water content of 0.15 g g^–1^), then carefully disintegrated into aggregates with a diameter smaller than 5 mm, homogenized, and packed into the plastic columns to obtain the same bulk density (1.1 g cm^–3^) in all columns. Columns were then wetted to a soil–water content of approximately 0.24 cm^3^cm^–3^. Three plant seeds were sown directly into the columns in a triangular spacing. Plants (three per each column) were initially irrigated with fresh water for 16 days. Next, plants in 16 soil columns were irrigated with a solution of CBZ and in eight columns with fresh water. The columns were weighed before and after irrigation to record the water balance and estimate evapotranspiration. An evaporation pan (diameter = 19.7 cm) was used to estimate the evapotranspiration demand in the greenhouse. Irrigation doses (Table S2) depended on decreases in average soil column water contents to keep plants’ optimal conditions. No outflow at the bottom was observed. The experiment was performed between May 15 and July 2, 2018, under greenhouse conditions (natural light, air humidity of 30%–40%, and air temperature of 20–24 °C).

The columns were analyzed as follows. One column was dissembled on the 16th day to examine fresh and dry masses of stems, leaves, and roots. Next samplings were carried out on the 23rd, 30th, 41st, and 48th day. Four columns were always analyzed to obtain information about a plant growth (i.e., the mass of roots, stems, leaves, and fruits, Figure S1), concentrations of CBZ and its metabolites in plant tissues, and concentrations of compounds in four soil layers. One column irrigated with fresh water was similarly analyzed as a control. Plant leaves were scanned, and their area (Figure S3) was evaluated using the ImageJ software, version 1.47.^30^ To monitor changes in the soil hydraulic properties over time, three undisturbed 100 cm^3^ soil samples were taken from one soil column and analyzed using the multistep outflow experiment.^31^ Soil water contents, θ (at depths of 2.5 and 15 cm), and pressure heads, *h* (at depths of 5 and 15 cm), were measured in four columns during the entire experiment using ECH_2_0 EC-5 sensors^32^ and microtensiometers Tensior 5,^33^ respectively. The Freundlich sorption isotherm and the dissipation half-live of CBZ in soil were evaluated using the batch sorption^34^ and degradation^35^ experiments, respectively. The resulting parameters are presented in Table 1.

**Table 1:** Model Parameters, Their Bounds, and Calculated 5%, 50%, and 95% Quantiles of the Parameters’ Posterior Distributions from the Bayesian Analysis^a^

			posterior distributions’ quantiles			
parameter^b^	parameter description	parameter bounds	5%	50%	95%	S1 [-]
Soil						
θ_*r*1_ [cm^3^cm^–3^]	residual water content	0.18				
θ_*s*1_ [cm^3^cm^–3^]	saturated water content	0.51				
α_1_ [cm^–1^]	VGM shape parameter	(0.001, 0.1)	0.009	0.013	0.016	0.11
*n*_1_ [-]	VGM shape parameter	(1.1, 3)	1.94	2.07	2.51	0.03
*K*_*s*1_ [cm day^–1^]	saturated hydraulic conductivity	70				
θ_*r*2_ [cm^3^cm^–3^]	residual water content	0.18				
θ_*s*2_ [cm^3^cm^–3^]	saturated water content	0.51				
α_2_ [cm^–1^]	VGM shape parameter	(0.001, 0.1)	0.015	0.022	0.035	0
*n*_2_ [−]	VGM shape parameter	(1.1, 3)	2.02	2.45	2.78	0
*K*_*s*2_ [cm day^–1^]	saturated hydraulic conductivity	70				
ρ*_b_* [g cm^–3^]	bulk density	1.1				
λ*_L_* [cm]	dispersivity	1				
*K_f_CBZ__* [cm^3β^ μg^1-β^ g^–1^]	soil–water partition coefficient	(1.1, 10)	2.24	2.44	2.63	0.07
β*_CBZ_* [-]	Freundlich exponent	0.89				
μ*_L_CBZ__* [day^–1^]	CBZ degradation rate in the liquid phase	0.0068				
μ*_S_CBZ__* [day^–1^]	CBZ degradation rate in the solid phase	0.0068				
*P0* [cm]	Feddes’ parameter	–15				
*POpt* [cm]	Feddes’ parameter	–30				
*P2H* [cm]	Feddes’ parameter	–300				
*P2L* [cm]	Feddes’ parameter	–500				
*P3* [cm]	Feddes’ parameter	–8000				
*C_max_^CBZ^* [g cm^–3^]	maximum CBZ concentration taken up by roots	(1 × 10^–9^, 1 × 10^–8^)	3.75 × 10^–9^	4.05 × 10^–9^	4.38 × 10^–9^	0.14
*C_max_^EPX^* [g cm^–3^]	maximum EPX concentration taken up by roots	(0, 1.0 × 10^–8^)	9.0 × 10^–12^	8.0 × 10^–11^	1.6 × 10^–10^	0
Roots						
*W* [cm^3^g_fw_^–1^]	root water content	0.88				
*K_RW_* [cm^3^g_fw_^–1^]	roots–water partition coefficient	(1, 30)	11.8	13.3	15.1	0.03
*M*^max^ [g_fw_]	maximum roots mass	308				
*M*^0^ [g_fw_]	minimum roots mass	15				
*K^gr^* [day^–1^]	root growth rate	0.2				
τ_R_ [day^–1^]	CBZ degradation rate in roots	(0, 0.55)	0.0	0.01	0.02	0.04
Stem						
*W* [cm^3^g_fw_^–1^]	stem water content	0.84				
*K_SW_* [cm^3^g_fw_^–1^]	stem–water partition coefficient	(1, 30)	10.5	11.8	12.8	0.06
*M*^max^ [g_fw_]	maximum stem mass	591				
*M*^0^ [g_fw_]	minimum stem mass	10				
*K^gr^* [day^–1^]	stem growth rate	0.14				
τ_S_ [day^–1^]	CBZ degradation rate in stem	(0, 0.55)	0.04	0.05	0.07	0.06
Leaves						
*W* [cm^3^g_fw_^–1^]	leaves water content	0.84				
*K_LW_* [cm^3^g_fw_^–1^]	leaves–water partition coefficient	(1, 30)	3.12	15.2	27.3	0
*M*^max^ [g_fw_]	maximum leaves mass	757				
*M*^0^ [g_fw_]	minimum leaves mass	14				
*K^gr^* [day^–1^]	leaves growth rate	0.13				
τ_L_ [day^–1^]	CBZ degradation rate in leaves	(0, 0.55)	0.38	0.44	0.50	0
*S_A_* [cm^2^g_fw_^–1^]	leaves specific area	70				
Fruits						
*W* [cm^3^g_fw_^–1^]	fruit water content	0.83				
*K_FW_* [cm^3^g_fw_^–1^]	fruit–water partition coefficient	(1, 30)	2.7	15.2	26.5	0
*M*^max^ [g_fw_]	Maximum fruit mass	720				
*M*^0^ [g_fw_]	Minimum fruit mass	0				
*K^gr^* [day^–1^]	fruit growth rate	0.65				
τ_F_ [day^–1^]	CBZ degradation rate in fruit	(0, 0.55)	0.0	0.02	0.04	0.08
*S_A_* [cm^2^g_fw_^–1^]	fruit specific area	50				
Compounds						
*m*_(CBZ)_ [g mol^–1^]	molar mass of CBZ	236.27				
*m*_(EPX)_ [g mol^–1^]	molar mass of EPX	252.28				

a The last column reports the first-order sensitivity indices (S1) calculated using the RBD-FAST method to assess the influence of different factors on the accumulation of CBZ in the edible fruits.

b The subscripts 1 and 2 indicate the first and second soil horizons, respectively.

### 2.2 Analytical Methods

Concentrations of compounds in soils and plant tissues were analyzed using the methods proposed and validated for CBZ and its four metabolites in our previous studies.^16,17,21,23,36^ Methods are also briefly described in the Supporting Information. Soils (four soil layers per each column) and plant tissues were freeze-dried and grounded. Compounds remaining in 2 g of soil samples were extracted with 4 mL of extraction mixture 1 (acetonitrile/water, 1/1, v/v, acidified with 0.1% of formic acid) followed by 4 mL of mixture 2 (acetonitrile/2-propanol/H_2_O, 3/3/4, v/v/v, acidified with 0.1% of formic acid) in an ultrasonic bath (DT 255, Bandelin Electronic, Sonorex Digitec, Berlin, Germany) for 15 min. Compounds in 0.05 g of plant tissue samples were extracted with 1 mL of extraction mixture 1 (acetonitrile/water, 1/1, 0.1% of formic acid) by shaking at 1800 min^–1^ for 5 min (TissueLyser II, Quiagen, Germany). A triple-stage quadrupole mass spectrometer, Quantiva (Thermo Fisher Scientific, San Jose, CA, USA), coupled with an Accela 1250 LC pump (Thermo Fisher Scientific) and HTS XT-CTC autosampler (CTC Analytics AG, Zwingen, Switzerland), was used for the analysis of irrigation water. A hybrid quadrupole-orbital trap mass spectrometer, Q Exactive HF Hybrid Quadrupole-Orbitrap Mass Spectrometer (Thermo Fisher Scientific, USA), operated in high-resolution product scan mode (HRPS), was used instead of a triple quadrupole for more complex soil and plant extracts. A Hypersil Gold aQ column (50 mm × 2.1 mm i.d., 5 μm particle size, from Thermo Fisher Scientific San Jose, CA, USA) was used for the chromatographic separation of these target compounds. A detailed description of the instrument settings can be found in ref (37). Recovery and LOQs of compounds in different matrices are presented in Tables S4 and S5, respectively.

### 2.3 Modeling Theory

#### 2.3.1 Model Description and Setup

The numerical model developed by Brunetti et al.^27^ is used to simulate the translocation and transformation of CBZ in the soil–plant continuum. The model combines the widely used hydrological model, HYDRUS-1D,^28^ with a multicompartment model of dynamic plant uptake.^38^ For a thorough description of the model, please refer to ref (27).

The Richards equation describes the variably saturated water flow in the soil:
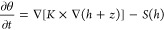
1where *t* is time [T], *z* is the vertical coordinate [L], θ is the volumetric water content [L^3^L^–3^], *K* is the unsaturated hydraulic conductivity [LT^–1^], *h* is the pressure head [L], and *S* is a sink term representing root water uptake [T^–1^]. The transport of the *i*th solute in the soil is described using the advection-dispersion-reaction equation, assuming that solutes can exist only in the solid and liquid phases:
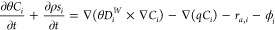
2where *C_i_* is the concentration of the *i*th solute in the liquid phase [ML^–3^], *s_i_* is the concentration of the *i*th solute in the solid phase [MM^–1^], ρ is the soil density [ML^–3^], *D*_*i*_^*W*^ is the dispersion tensor for the *i*th solute in water [L^2^T^–1^], *q* is the water flux [LT^–1^], *r_a,I_* is the root solute uptake term [ML^–3^ T^–1^], and ϕ*_i_* represents the reaction sink/source term [ML^–3^ T^–1^].

The soil column profile (a depth of 20 cm) is discretized in 100 finite elements refined at the top to accommodate gradients induced by the atmospheric conditions. The soil domain is divided into two horizons (0 ≤ *z* < −10 cm; −10 ≤ *z* ≤ −20 cm) to account for the effect of different root densities (Figure S2) on soil hydraulic properties, which are described by the unimodal van Genuchten–Mualem (VGM) function^39^ (parameters: θ*_r_* [L^3^L^–3^], θ*_s_* [L^3^L^–3^], α [L^–1^], *n* [-], and *K_s_* [LT^–1^]). The batch experiment data, presented by Schmidtová et al.,^34^ indicates that the Freundlich adsorption isotherm can describe the sorption of CBZ to the solid phase (parameters: *K_f_CBZ__* [L^3β^ M^1-β^ M^–1^] and β*_CBZ_* [-]). Measured data show that carbamazepine 10,11-epoxide (EPX) is the only CBZ metabolite detected in the soil during the experiment (Table S7). The CBZ degradation process in the soil is thus simulated using a sequential decay chain with first-order rate coefficients, μ*_L_CBZ__* and μ*_S_CBZ__* [T^–1^], accounting for the transformation of CBZ in EPX in the liquid and solid phases, respectively.

The measured water content at the beginning of the experiment is used as the initial condition. No solute was present in the soil before starting the experiment. An atmospheric boundary condition is applied at the soil surface, while a seepage face boundary condition (BC) is set at the bottom. The concentration flux across the top boundary is simulated using the classic Cauchy-type BC, while a zero concentration gradient is imposed at the bottom.

The piecewise linear Feddes function parameterizes the root water stress^40^ and regulates actual transpiration (parameters: *P0*, *POpt*, *P2H*, *P2L*, and *P3*). Simultaneously, a root density function modulates actual root water uptake depending on the root growth. A logistic growth function was fitted to the measured wet masses of roots at different depths (Figure S2) to determine the average root growth rate, which was then used to estimate the root density during the numerical simulation. The measured evapotranspiration demand is partitioned into potential evaporation and transpiration using a time series of leaf area indices, which were calculated by fitting a logistic function to the measured one-sided leaves area (Figure S3). The difference between total evapotranspiration, measured by weighing the columns, and transpiration is used to estimate evaporation from the soil surface (Figure S4). All compounds dissolved in water are passively taken up by plant roots when their concentrations are lower than maximum allowed solution concentrations, *C_max_* [ML^–3^].^41^

Plants are conceptualized into four compartments: roots, stem, leaves, and fruits. Each compartment is assumed to have a logistic growth^38^ (parameters: *W* [L^3^M^–1^], *M*^max^ [M], *M*^0^ [M], *K^gr^* [T^–1^], and *S_A_* [L^2^M^–1^]). The specific areas of fruits and leaves are used to partition transpiration fluxes. The CBZ solution was carefully applied on the soil surface to minimize its dispersion in the surrounding environment. Therefore, the effect of gaseous uptake and particle deposition could be neglected in the model. Similarly, volatilization is excluded from modeling due to the nonvolatile behavior of CBZ. Tissue-water partitioning (*K_RW_*, *K_SW_*, *K_LW_*, and *K_FW_* [L^3^M^–1^]) is assumed. Since the only detectable CBZ’s metabolite in plants was EPX (Table S6), the analysis was restricted to these two compounds using a first-order degradation coefficient, τ [T^–1^], in each compartment, which simulates the transformation of CBZ in EPX. While input concentrations and sorption parameters are converted to molar units before executing the model, output concentrations are reported in ng/g for visualization purposes.

#### 2.3.2 Bayesian Analysis

This study adopts a probabilistic approach based on the Bayesian inference to calibrate the dynamic plant uptake model and assess its predictive uncertainty. Model calibration is required to estimate parameters that are difficult to measure in the laboratory (e.g., degradation rates)^3^ and others (e.g., VGM parameters) that need to be adjusted to match the experimental conditions, which can be different from those encountered in the laboratory.^42^ To this aim, the multimodal nested sampling (MULTINEST)^43^ is combined with the soil–plant model and measured data to estimate the parameters’ posterior distribution. Multiple studies have demonstrated the accuracy and computational efficiency of this algorithm,^44−47^ whose thorough description can be found in refs (43) and (48). As in refs (44) and (45), a convergence analysis is used to assess the stability and accuracy of the MULTINEST estimator.

If we assume that error residuals are uncorrelated and normally distributed with a constant variance, σ^2^, the log-likelihood function  for the *j*th set of measurements can be written as
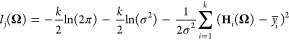
3where **H***_i_*(**Ω**) and *y̅_i_* are the *i*th model realization and its corresponding measured value, respectively, and *k* is the number of observations in the *j*th set of measurements. The measured data used in the Bayesian analysis include the time series of volumetric water contents (θ) and pressured heads (*h*) at two different locations, CBZ (*C_soil,CBZ_*) and EPX (*C_S,EPX_*) concentrations in the soil and CBZ and EPX concentrations in the roots (*C_R,CBZ_*, *C_R,EPX_*), stem (*C_S,CBZ_*, C*_S,EPX_*), leaves (*C_L,CBZ_*, *C_L,EPX_*), and fruits (*C_F,CBZ_*, *C_F,EPX_*). Therefore, the log-likelihood function *L(***Ω**) is the aggregated sum of single likelihoods. The mean and variance of each measurement set used in the model calibration are calculated from the column replicates (Tables S6 and S7).
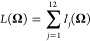
4

The dimensionality of the inverse problem is reduced by fixing specific model parameters (Table 1). In particular, the results of multistep outflow experiments are used to fix the residual and saturated water contents and saturated hydraulic conductivity. This choice is motivated by the dynamics of the measured soil moisture, which never approached nearly saturated conditions during the experiment, thus providing negligible information content in that range. Feddes’ parameters for green peas (i.e., *P0*, *POpt*, *P2H*, *P2L*, and *P3* in Table 1) are taken from the literature.^49^ Results of batch and degradation experiments are used to fix the Freundlich exponent and the first-order degradation coefficients for CBZ, respectively. The same sorption parameters are assumed for EPX, whose further degradation in the soil is neglected.^21^ Water contents of plants’ tissues are assumed constant and calculated from the average measured fresh and dry weights during the experiment. Growth parameters of different plant compartments are estimated by fitting logistic functions to the time series of measured plant masses (Figures S1 and S2). Due to these prior assumptions, the dimensionality of the problem is reduced to 15 parameters. Uniform prior distributions are used in the Bayesian analysis. Parameter bounds are provided in Table 1.

#### 2.3.3 Bioaccumulation of Carbamazepine in Edible Fruits: Global Sensitivity Analysis

After the model calibration and the uncertainty assessment, a Global Sensitivity Analysis is performed on the calibrated parameters to identify the most important factors driving the bioaccumulation of CBZ in green peas’ fruits. The analysis provides a statistical basis to shed light on the relative importance of different physicochemical processes on the translocation of CBZ toward the edible parts of the plant, which are directly linked to human and animals’ intake.

The Random Balance Designs Fourier Amplitude Sensitivity Test (RBD-FAST)^50,51^ is applied. The method combines the accuracy of the classic Fourier Amplitude Sensitivity Test^52^ with the computational cheapness of Satterthwaite’s random balance designs^53^ to estimate the first-order effect (S1) in a variance-based context.^54^ Higher S1 values are attributed to more influential parameters. The main advantage of the RBD-FAST method is that the total number of model runs is kept down to *N* instead of *d* × *N* (like in Sobol’ or FAST methods), where *d* is the number of factors investigated. In the present study, a convergence analysis is used to determine *N* (Figure S5).

## 3 Results and Discussion

### 3.1 Bayesian Analysis

**Figure 1:**
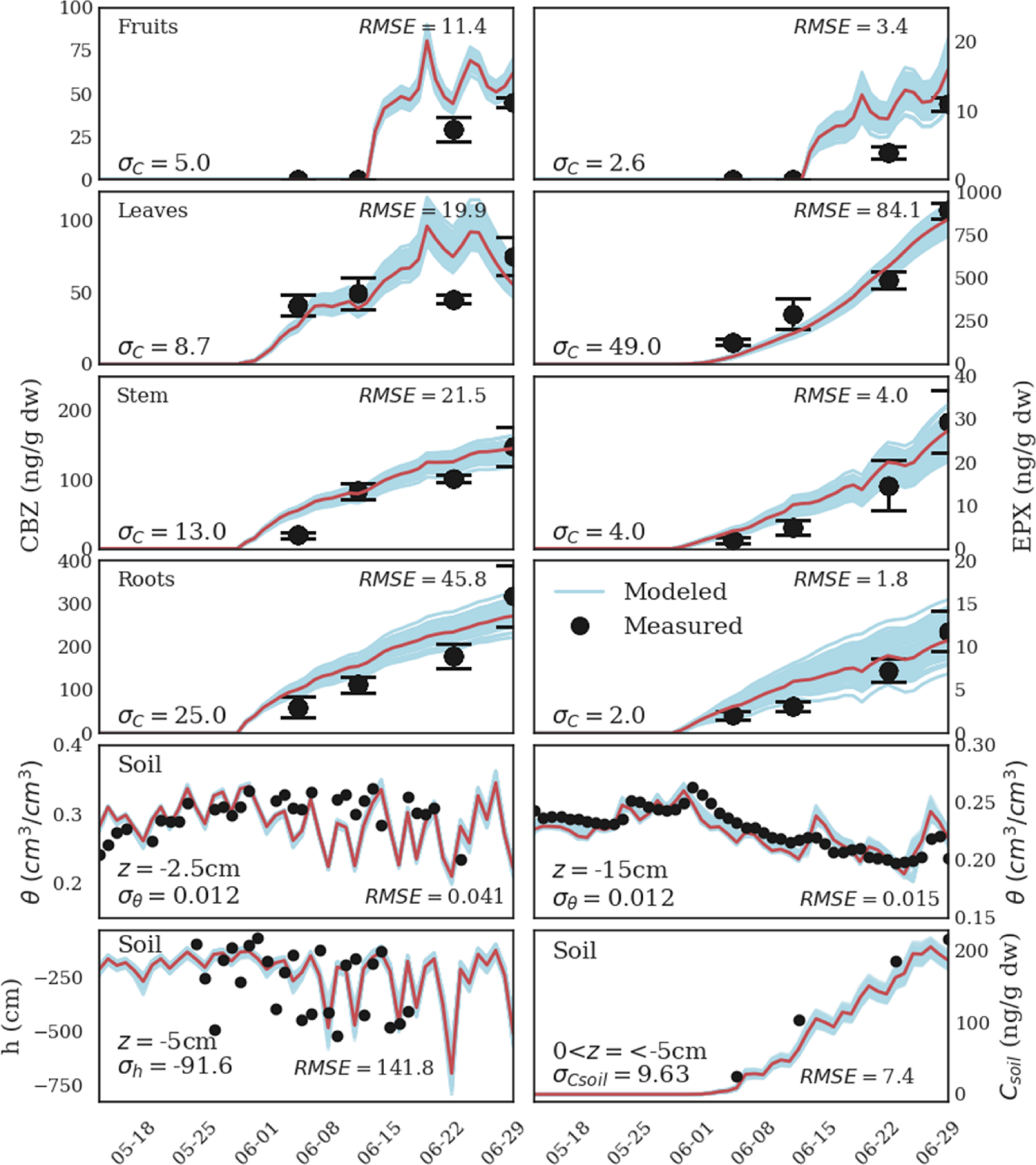
Comparison between the measured (black circles) CBZ (left column) and EPX (right column) concentrations in different plant’s compartments, CBZ concentration in soil (0 < *z* < −5 cm), pressure heads (*z* = −5 cm), and volumetric water contents (*z* = −2.5 and −15 cm), and corresponding modeled values (blue lines) obtained by random sampling of 100 solutions from the posterior parameter distributions. The error bars indicate the standard deviations of the measurements. The red line indicates the model predictions obtained by using the median solution reported in Table 1. The mean standard deviation used in the Bayesian analysis (σ) and the median root mean square error (RMSE) are reported in each subplot.

Figure 1 shows a comparison between the measured (black circles) CBZ concentrations in different plant’s compartments and soil (0 < *z* < −5 cm), pressure heads (*z* = −5 cm), and volumetric water contents at two different depths (*z* = −2.5 and 15 cm) and the model predictive checks (blue lines) obtained by random sampling of 100 solutions from the posterior parameter distributions. Posterior predictive checks are used to assess the effect of the uncertainty on the model predictions and to “look for systematic discrepancies between real and simulated data”,^55^ thus representing a fundamental tool for model criticism. Results demonstrate that the model can reproduce water flow and solute accumulation in the soil as well as the dynamics of the translocation and metabolization of CBZ and EPX in all plant compartments, with limited uncertainty and satisfactory accuracy. The highest discrepancy is observed in the fruit and leaves compartments, for which the model overestimates the solute concentrations, though the error magnitude is low. This deviation can be attributed to dynamic variations in the plant water content and the sorption coefficient during the experiment, which are not considered in the model. The volumetric water content is well described, while the model seems to underestimate the variance in the measured pressure head. However, this tendency might be partially related to a very small measurement footprint of the tensiometers, which makes them extremely sensitive to heterogeneities in the soil’s hydraulic regime not represented in the model.

The calculated 5%, 50%, and 95% quantiles of the parameters’ posterior distributions are reported in Table 1 and indicate a well-posed inverse problem characterized by low parameters’ uncertainty. The difference between the VGM shape parameters for the two soil horizons is appreciable and confirms the roots’ potential influence on water flow. In particular, smaller values of α and *n* calculated for the first soil layer are compatible with an increased pore heterogeneity caused by a higher root density in that part of the soil column.^56^ The estimated soil–water partition coefficient, *K_f_CBZ__*, is in line with the results of the batch experiment (i.e., *K_f_CBZ__* ≈ 2.8 cm^3β^ μg^1-β^ g^–1^) and with other studies,^27,34^ thus confirming the overall reliability of the analysis. The narrow confidence intervals estimated for the soil hydraulic parameters suggest that the soil behavior influences the uptake and translocation processes. This aspect is further investigated in the following sensitivity analysis.

Interestingly, the Bayesian calibration framework reveals a limited solute uptake capacity of plants (Table 1). This is also confirmed by the measured data, which show a much higher persistence of CBZ in the soil than in the plants (Figure S7). In particular, the CBZ and EPX median maximum concentrations taken up by plants are 4.45 and 0.1 ng cm^–3^, respectively. The reduced uptake mechanism is potentially explained by the difference between root permeability to chemicals and water, making the uptake of CBZ and EPX slower than that of water. The use of a maximum uptake concentration used in HYDRUS-1D shares some conceptual and mathematical similarities with the retardation factor proposed by Gredelj et al.^57^ to simulate the translocation of perfluoroalkyl acids in red chicory. However, this model parameter lacks physical meaning and theoretical background to generalize it for other modeling circumstances. The difference between *C_max_^CBZ^* and *C_max_^EPX^* suggests that CBZ is taken up to a greater extent than EPX, whose occurrence in plant tissues results from in-plant metabolism rather than plant uptake. This is also confirmed by the negligible amount of EPX observed in the soil (Table S7).

Comparing the estimated first-order degradation rates further reveals that most of the CBZ metabolization occurs in the leaves. This is further highlighted by the significantly higher EPX concentrations measured in the leaves compared to other compartments. In contrast, roots and stem mainly act as reservoirs for CBZ, which is either sorbed or translocated upward. This behavior was observed in other studies as well.^2,16,17,20,23,25,27^ Both the experiment and the model indicate a low accumulation of a chemically active compound, such as EPX, in the plant’s edible part. The lower accumulation of both compounds in fruits can be partially explained by the significantly shorter exposure to the contamination and lower transpiration of fruits than leaves.^23^ In addition, the metabolization of CBZ in fruits has been proven to be generally less efficient.^8,19,23^

The comparison between the estimated partition coefficients confirms that sorption occurs mainly in the roots and stem, while the wide confidence intervals for *K_LW_* and *K_FW_* imply the lack of their statistical identifiability. The estimated median value for roots (i.e., *K_RW_* = 13.3 cm^3^g_fw_^–1^) agrees with Chuang et al.^1^ and is mainly related to the affinity of CBZ to the plant composition^58^ and its hydrophobicity.^59^ In particular, different plant components such as lipids, carbohydrates, waxes, lignin, and suberin can act as sorption sites for chemicals.^60^ Their distribution is tissue-dependent,^61^ thus leading to different sorption coefficients in different plant compartments.

### 3.2 Global Sensitivity Analysis

The convergence analysis indicated that 3000 model executions were sufficient to obtain stable estimations of the first-order sensitivity indices (reported in the last column of Table 1) for all investigated parameters (Figure S5). Nine factors out of 15 exhibit an appreciable influence on the translocation of CBZ toward fruits. The most influential parameter is the maximum concentration taken up by plants, which regulates the solute amount entering the roots. Once the compound enters the plant, the sorption and degradation processes in the roots and stem influence its translocation into the fruits, as suggested by the calculated sensitivity indices. Higher sorption in these compartments retards the translocation of CBZ in the transpiration streams and provides more time for the in-place enzymatic degradation. On the other hand, the leaves’ role is insignificant as the phloem flux is neglected in the numerical simulations.

**Figure 2:**
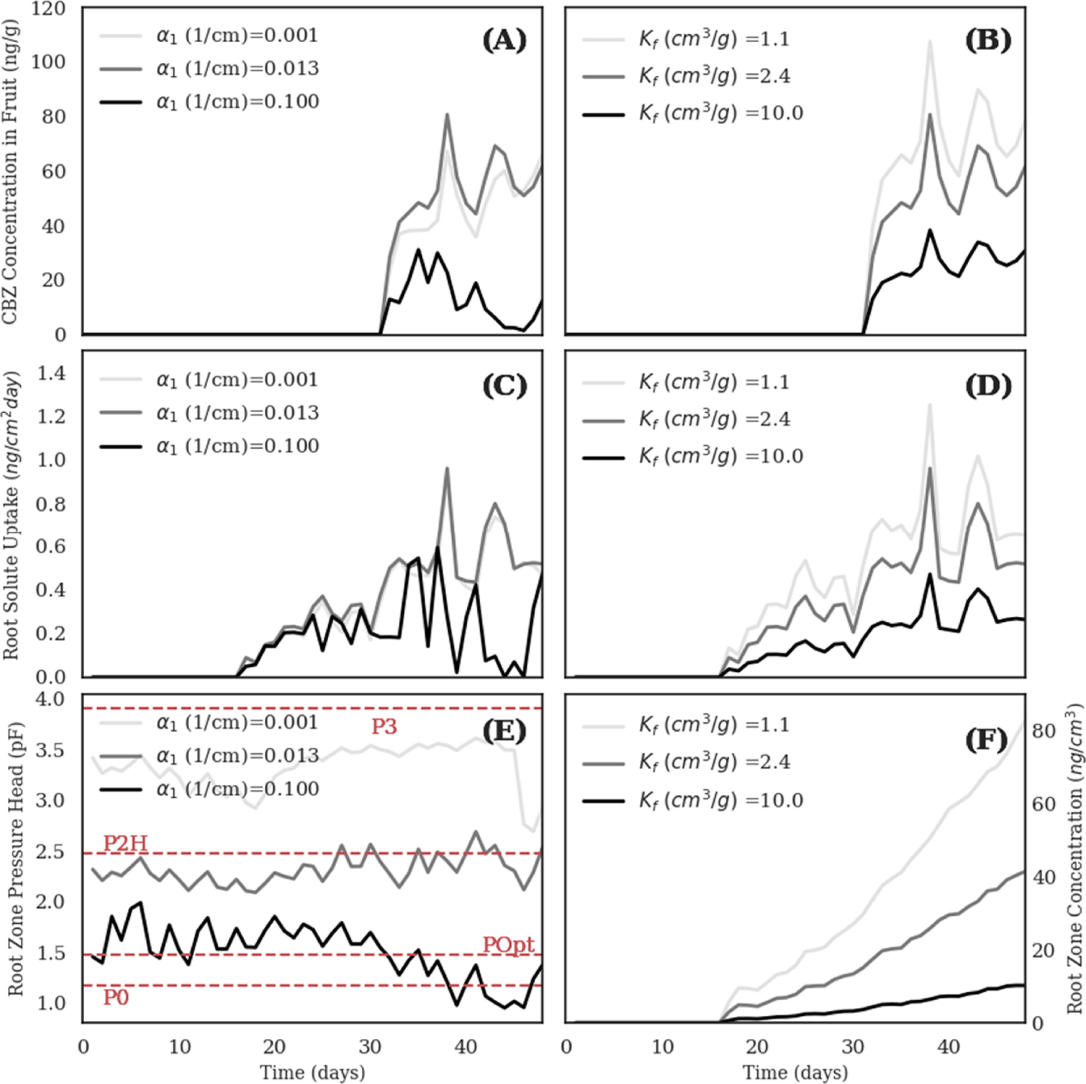
Sensitivity analysis results. (A) and (B) Simulated CBZ concentrations in the fruits as a function of α and *K_f_*, respectively. (C) and (D) Simulated root solute uptake as a function of α and *K_f_*, respectively. (E) Simulated average pressure head (*pF* = log(|*h*|) in the root zone as a function of α. (F) Simulated average CBZ concentrations in the liquid phase in the root zone as a function of *K_f_*. The red dashed lines indicate the Feddes’ parameters (Table 1). The plots were obtained by performing numerical simulations with three different values of α_1_ and *K_f_*. In particular, the median solution reported in Table 1 was used as a reference, and only the values of α_1_ and *K_f_* were alternatively changed to match the minimum and the maximum listed in Table 1.

The role of soil hydraulic properties holds more interest. The sensitivity analysis reveals that the VGM shape parameters of the first soil horizon and the soil–water partition coefficient strongly influence the accumulation of CBZ in the fruit compartment. In contrast, the contribution of the second soil layer is insignificant. The roots and solute distribution in the soil are both contributing factors to this behavior. Root water and solute uptake occurs mainly in the upper part of the soil profile, where the root density is higher, and CBZ is more bioavailable due to the nonlinear sorption process (Figure S6). The solute movement toward deeper soil horizons is restricted by low water flow velocities and solute dispersivity. Under such conditions, the air-entry pressure parameter α_1_ partially regulates actual root CBZ uptake (Figure 2), the CBZ concentration in the fruit compartment, and the root zone pressure head and concentration, which increase with increasing values of α_1_ and *K_f_*, respectively. Root solute uptake is negatively correlated with α_1_ [(C) in Figure 2], mainly due to the change in the transpiration pattern induced by different pressure head distributions in the root zone [(E) in Figure 2]. In the simulated scenarios, higher values of α_1_ lead to a lower (in absolute value) simulated root zone pressure head, which drops below the optimal pressure head value for transpiration (i.e., *POpt* in the Feddes’ model), thus reducing actual root water and solute uptake. This physical behavior is exacerbated after *t* = 30 days [(C) and (E) in Figure 2] and results in lower translocation of CBZ toward the fruit compartment [(A) in Figure 2]. Conversely, for α_1_ = 0.001 (1/cm), the root zone pressure head is shifted toward much higher values, where the actual transpiration rate is also reduced, though the effect is less pronounced. For α_1_ = 0.013 (1/cm) (i.e., median value in Table 1), the root zone pressure head oscillates in the optimal transpiration range (i.e., *P2H* < *h* < *POpt*). Therefore, it can be concluded that soil hydraulic properties affect the accumulation of CBZ in fruits by simultaneously driving water flow in the unsaturated domain and modulating the transpiration pattern. These effects then propagate nonlinearly into the plant. However, it must be emphasized that these conclusions are not general but restricted to the modeling scenario investigated in the present study.

On the other hand, CBZ sorption in the soil affects linearly simulated CBZ concentrations in the fruit compartment. Generally, higher sorption (which for CBZ increases with increasing organic matter content in soils^34^) reduces the solute availability in the root zone [(F) in Figure 2], thus reducing the amount of solute entering the roots [(F) in Figure 2] and the concentration in the fruits [(B) in Figure 2]. Klement et al.^23^ found positive correlations between *K_f_* and normalized concentration loads of the parent compound CBZ and its metabolites. In particular, the correlation was significant (95% level) for roots and stems but not significant for leaves and fruits. This suggests that organic-rich soils might reduce the translocation of CBZ and similar compounds to the edible parts of the plant.

### 3.3 Strengths and Limitations

We consider this study to be innovative in two main aspects:

To our knowledge, this is the first study involving numerical simulations of dynamic plant uptake of chemicals that includes extensive spatial and temporal measurements of soil- and plant-related quantities. Indeed, compared to previous studies,^62^ the dataset includes measured volumetric water contents and soil pressure heads at different depths, providing further information about the unsaturated soil domain. This significant and variegated amount of data allows us to characterize water flow and reactive solute transport in the soil–plant continuum with low predictive uncertainty and satisfactory accuracy.
Compared to other inverse modeling studies,^44,63−66^ this study applies, for the first time, a Bayesian calibration framework to estimate both soil and plant parameters, thus providing a statistical basis for the uncertainty assessment in the soil–plant continuum. Furthermore, using the Nested Sampling algorithm allows us to estimate the posterior parameter distribution and the marginal likelihood simultaneously. While not being of immediate interest to the present study, the latter can be used to assess model complexity, a valuable tool when dealing with highly complex and parameterized models. This methodological feature represents an advantage against other approaches focused on the use of Monte Carlo Markov Chain algorithms.^63−65^

However, these strengths are paired with several limitations. First, for most practical risk assessment applications, it is not possible to obtain comprehensive measurements due to time constraints and budget limitations. This raises some questions regarding the model’s applicability in a predictive setting in case of limited observations. There is no clear and definite answer to this. Still, we advocate the ubiquitous use of uncertainty analysis (e.g., Bayesian analysis) to identify the most justifiable model complexity level in light of the information content of the data available. Furthermore, a Global Sensitivity Analysis can, as demonstrated in this study, be used in a prognostic way to target the most important processes and focus the experimental and modeling effort on them. In such circumstances, a cross-disciplinary approach is of crucial importance.

Other model limitations include the macroscopic description of root water uptake and the lack of the physical meaning of the maximum uptake concentration. The former simplifies the root system hydraulics, which plays an important role in root water and solute uptake.^67^ The latter hinders the generalization of the particular modeling approach, which is appealing from a computational point of view due to its simplicity, to other conditions. Therefore, it is important to improve the representation of these processes in the model to increase its generalizability and accuracy.

### 3.4 Implications and Future Outlook

This study’s main aim was to evaluate further the performance of the coupled soil–plant model developed by Brunetti et al.^27^ against measurements from a comprehensive experiment on the translocation and transformation of CBZ in green pea plants in a controlled environment. A probabilistic Bayesian framework was used to calibrate the model and assess its predictive uncertainty. Results confirmed that the model could reproduce accurately and with low uncertainty the transport of CBZ in the soil and its translocation and transformation in different plant tissues. The following Global Sensitivity Analysis has further highlighted the role of soil in bioaccumulation of CBZ in the fruit compartment, mainly by regulating actual transpiration streams and CBZ bioavailability in the root zone.

Overall, the study confirms that the model can be successfully used for simulating the translocation of neutral compounds in the soil–plant continuum in a partially mechanistic way. This opens new possibilities for a more comprehensive assessment of the contamination risk, allowing the development of reliable mitigation strategies for environmental pollution problems in both the soil and plant domains. On this basis, future studies should explore the possibility of extending the model applicability to ionic compounds frequently encountered in contaminated sites. However, this would require extensive modifications to the plant model.^68^

## Supporting Information Available

Detailed description of the analytical methods, (Table S1) basic soil characteristics of the Haplic Chernozem on loess, (Table S2) irrigation doses and carbamazepine concentrations, (Table S3) information about LC–MS/MS parameters, (Table S4) compound recovery in 200 ng per 2 g of soil and 50 ng per 0.05 g of plant tissues (%), (Table S5) limits of compounds’ quantification (ng g^–1^), (Table S6) concentrations of carbamazepine and carbamazepine 10,11-epoxide in plant tissues, (Table S7) concentrations of carbamazepine and carbamazepine 10,11-epoxide in soils, (Figure S1) total wet and dry masses and water ratios of plant tissues in each column, (Figure S2) wet and dry masses of roots at different soil depths, (Figure S3) total area of leaves in each column evaluated on scanned fresh leaves using the ImageJ software, (Figure S4) measured transpiration, evaporation, and cumulative evapotranspiration, (Figure S5) convergence analysis of first-order sensitivity indices, (Figure S6) simulated root water uptake, pore water concentration, and root solute uptake along the soil profile at different times, and (Figure S7) measured average fractions of the applied solute mass (the sum of CBZ and EPX) in the soil and different plant compartments at different times (PDF)

## References

[ref1] Chuang Y.-H.; Liu C.-H.; Sallach J. B.; Hammerschmidt R.; Zhang W.; Boyd S. A.; Li H. Mechanistic Study on Uptake and Transport of Pharmaceuticals in Lettuce from Water. Environ. Int. 2019, 131, 104976 10.1016/j.envint.2019.104976. 31336255

[ref2] Goldstein M.; Malchi T.; Shenker M.; Chefetz B. Pharmacokinetics in Plants: Carbamazepine and Its Interactions with Lamotrigine. Environ. Sci. Technol. 2018, 52, 6957 10.1021/acs.est.8b01682. 29787250

[ref3] Hurtado C.; Trapp S.; Bayona J. M. Inverse Modeling of the Biodegradation of Emerging Organic Contaminants in the Soil-Plant System. Chemosphere 2016, 156, 236 10.1016/j.chemosphere.2016.04.134. 27179241

[ref4] Li Y.; Chuang Y. H.; Sallach J. B.; Zhang W.; Boyd S. A.; Li H. Potential Metabolism of Pharmaceuticals in Radish: Comparison of *in Vivo* and *in Vitro* Exposure. Environ. Pollut. 2018, 242, 962 10.1016/j.envpol.2018.07.060. 30373041

[ref5] Malchi T.; Maor Y.; Tadmor G.; Shenker M.; Chefetz B. Irrigation of Root Vegetables with Treated Wastewater: Evaluating Uptake of Pharmaceuticals and the Associated Human Health Risks. Environ. Sci. Technol. 2014, 42, 9325 10.1021/es5017894. 25026038

[ref6] Zhang H.; Chen J.; Ni Y.; Zhang Q.; Zhao L. Uptake by Roots and Translocation to Shoots of Polychlorinated Dibenzo-*p*-Dioxins and Dibenzofurans in Typical Crop Plants. Chemosphere 2009, 76, 740 10.1016/j.chemosphere.2009.05.030. 19541345

[ref7] Bhalsod G. D.; Chuang Y.-H.; Jeon S.; Gui W.; Li H.; Ryser E. T.; Guber A. K.; Zhang W. Uptake and Accumulation of Pharmaceuticals in Overhead- and Surface-Irrigated Greenhouse Lettuce. J. Agric. Food Chem. 2018, 66, 822 10.1021/acs.jafc.7b04355. 29293328

[ref8] Riemenschneider C.; Seiwert B.; Moeder M.; Schwarz D.; Reemtsma T. Extensive Transformation of the Pharmaceutical Carbamazepine Following Uptake into Intact Tomato Plants. Environ. Sci. Technol. 2017, 51, 6100 10.1021/acs.est.6b06485. 28506063

[ref9] So E. L.; Ruggles K. H.; Cascino G. D.; Ahmann P. A.; Weatherford K. W. Seizure Exacerbation and Status Epileptics Related to Carbamazepine-10,11-epoxide. Ann. Neurol. 1994, 35, 743 10.1002/ana.410350616. 8210232

[ref10] Warner T.; Patsalos P. N.; Prevett M.; Elyas A. A.; Duncan J. S. Lamotrigine-Induced Carbamazepine Toxicity: An Interaction with Carbamazepine-10,11 -Epoxide. Epilepsy Res. 1992, 11, 147 10.1016/0920-1211(92)90049-Y. 1618180

[ref11] Eugenio N. R.; McLaughlin M.; Pennock D. Soil Pollution: A Hidden Reality.; FAO: 2018.

[ref12] Charuaud L.; Jarde E.; Jaffrezic A.; Thomas M. F.; Le Bot B. Veterinary Pharmaceutical Residues from Natural Water to Tap Water: Sales, occurrence and fate. J. Hazard. Mater. 2019, 361, 169 10.1016/j.jhazmat.2018.08.075. 30179788

[ref13] Picó Y.; Alvarez-Ruiz R.; Alfarhan A. H.; El-Sheikh M. A.; Alshahrani H. O.; Barceló D. Pharmaceuticals, Pesticides, Personal Care Products and Microplastics Contamination Assessment of Al-Hassa Irrigation Network (Saudi Arabia) and Its Shallow Lakes. Sci. Total Environ. 2020, 701, 135021 10.1016/j.scitotenv.2019.135021. 31734487

[ref14] Verlicchi P.; Zambello E. Pharmaceuticals and Personal Care Products in Untreated and Treated Sewage Sludge: Occurrence and Environmental Risk in the Case of Application on Soil - A Critical Review. Sci. Total Environ. 2015, 538, 750 10.1016/j.scitotenv.2015.08.108. 26327643

[ref15] Kumar K.; Gupta S. C. A Framework to Predict Uptake of Trace Organic Compounds by Plants. J. Environ. Qual. 2016, 45, 555 10.2134/jeq2015.06.0261. 27065403

[ref16] Kodešová R.; Klement A.; Golovko O.; Fér M.; Nikodem A.; Kočárek M.; Grabic R. Root Uptake of Atenolol, Sulfamethoxazole and Carbamazepine, and Their Transformation in Three Soils and Four Plants. Environ. Sci. Pollut. Res. 2019, 26, 9876 10.1007/s11356-019-04333-9. 30734257

[ref17] Kodešová R.; Klement A.; Golovko O.; Fér M.; Kočárek M.; Nikodem A.; Grabic R. Soil Influences on Uptake and Transfer of Pharmaceuticals from Sewage Sludge Amended Soils to Spinach. J. Environ. Manage. 2019, 250, 109407 10.1016/J.JENVMAN.2019.109407. 31472377

[ref18] Zhang Y.; Geißen S. U.; Gal C. Carbamazepine and Diclofenac: Removal in Wastewater Treatment Plants and Occurrence in Water Bodies. Chemosphere 2008, 73, 1151 10.1016/j.chemosphere.2008.07.086. 18793791

[ref19] Shenker M.; Harush D.; Ben-Ari J.; Chefetz B. Uptake of Carbamazepine by Cucumber Plants - A Case Study Related to Irrigation with Reclaimed Wastewater. Chemosphere 2011, 82, 905 10.1016/j.chemosphere.2010.10.052. 21071061

[ref20] Dordio A. V.; Belo M.; Martins Teixeira D.; Palace Carvalho A. J.; Dias C. M. B.; Picó Y.; Pinto A. P. Evaluation of Carbamazepine Uptake and Metabolization by Typha Spp., a Plant with Potential Use in Phytotreatment. Bioresour. Technol. 2011, 102, 7827 10.1016/j.biortech.2011.06.050. 21745739

[ref21] Koba O.; Golovko O.; Kodešová R.; Klement A.; Grabic R. Transformation of Atenolol, Metoprolol, and Carbamazepine in Soils: The Identification, Quantification, and Stability of the Transformation Products and Further Implications for the Environment. Environ. Pollut. 2016, 218, 574 10.1016/j.envpol.2016.07.041. 27514306

[ref22] Kodešová R.; Kočárek M.; Klement A.; Golovko O.; Koba O.; Fér M.; Nikodem A.; Vondráčková L.; Jakšík O.; Grabic R. An Analysis of the Dissipation of Pharmaceuticals under Thirteen Different Soil Conditions. Sci. Total Environ. 2016, 544, 369 10.1016/j.scitotenv.2015.11.085. 26657382

[ref23] Klement A.; Kodešová R.; Golovko O.; Fér M.; Nikodem A.; Kočárek M.; Grabic R. Uptake, Translocation and Transformation of Three Pharmaceuticals in Green Pea Plants. J. Hydrol. Hydromech. 2020, 68, 1 10.2478/johh-2020-0001.

[ref24] Montemurro N.; Postigo C.; Lonigro A.; Perez S.; Barceló D. Development and Validation of an Analytical Method Based on Liquid Chromatography–Tandem Mass Spectrometry Detection for the Simultaneous Determination of 13 Relevant Wastewater-Derived Contaminants in Lettuce. Anal. Bioanal. Chem. 2017, 409, 5375 10.1007/s00216-017-0363-1. 28493020

[ref25] Goldstein M.; Shenker M.; Chefetz B. Insights into the Uptake Processes of Wastewater-Borne Pharmaceuticals by Vegetables. Environ. Sci. Technol. 2014, 48, 5593 10.1021/es5008615. 24749778

[ref26] Brunetti G.; Šimůnek J.; Glöckler D.; Stumpp C. Handling model complexity with parsimony: Numerical analysis of the nitrogen turnover in a controlled aquifer model setup. J. Hydrol. 2020, 584, 124681 10.1016/j.jhydrol.2020.124681.

[ref27] Brunetti G.; Kodešová R.; Šimůnek J. Modeling the Translocation and Transformation of Chemicals in the Soil-Plant Continuum: A Dynamic Plant Uptake Module for the HYDRUS Model. Water Resour. Res. 2019, 55, 8967 10.1029/2019WR025432.

[ref28] Šimůnek J.; van Genuchten M. T.; Šejna M. Recent Developments and Applications of the HYDRUS Computer Software Packages. Vadose Zone J. 2016, 15, 1–25. 10.2136/vzj2016.04.0033.

[ref29] Trapp S. Fruit Tree Model for Uptake of Organic Compounds from Soil and Air. SAR QSAR Environ. Res. 2007, 18, 367–387. 10.1080/10629360701303693. 17514576

[ref30] Schneider C. A.; Rasband W. S.; Eliceiri K. W. NIH Image to ImageJ: 25 Years of Image Analysis. Nat. Methods 2012, 9, 671–675. 10.1038/nmeth.2089. 22930834 PMC5554542

[ref31] van Dam J. C.; Stricker J. N. M.; Droogers P. Inverse Method to Determine Soil Hydraulic Functions from Multistep Outflow Experiments. Soil Sci. Soc. Am. J. 1994, 58, 647–652. 10.2136/sssaj1994.03615995005800030002x.

[ref32] METER Group Inc. USA. EC-5 Moisture Sensor, User Manual; Decagon Devices, Inc.: 2018.

[ref33] METER Group Inc. USA. T5/T5X Pressure Transducer Tensiometer, User Manual Version 8/2018; METER Group: 2018.

[ref34] Schmidtová Z.; Kodešová R.; Grabicová K.; Kočárek M.; Fér M.; Švecová H.; Klement A.; Nikodem A.; Grabic R. Competitive and Synergic Sorption of Carbamazepine, Citalopram, Clindamycin, Fexofenadine, Irbesartan and Sulfamethoxazole in Seven Soils. J. Contam. Hydrol. 2020, 234, 103680 10.1016/j.jconhyd.2020.103680. 32682147

[ref35] Kodešová R.; Chroňáková A.; Grabicová K.; Kočárek M.; Schmidtová Z.; Frková Z.; Vojs Staňová A.; Nikodem A.; Klement A.; Fér M.; Grabic R. How Microbial Community Composition, Sorption and Simultaneous Application of Six Pharmaceuticals Affect Their Dissipation in Soils. Sci. Total Environ. 2020, 764, 141134 10.1016/j.scitotenv.2020.141134. 32768780

[ref36] Golovko O.; Koba O.; Kodesova R.; Fedorova G.; Kumar V.; Grabic R. Development of Fast and Robust Multiresidual LC-MS/MS Method for Determination of Pharmaceuticals in Soils. Environ. Sci. Pollut. Res. 2016, 23, 14068–14077. 10.1007/s11356-016-6487-6. 27044290

[ref37] Grabicova K.; Vojs Staňová A.; Koba Ucun O.; Borik A.; Randak T.; Grabic R. Development of a Robust Extraction Procedure for the HPLC-ESI-HRPS Determination of Multi-Residual Pharmaceuticals in Biota Samples. Anal. Chim. Acta 2018, 1022, 53 10.1016/j.aca.2018.04.011. 29729738

[ref38] Trapp S. Fruit Tree Model for Uptake of Organic Compounds from Soil and Air. SAR QSAR Environ. Res. 2007, 18, 367 10.1080/10629360701303693. 17514576

[ref39] van Genuchten M. T. A Closed-Form Equation for Predicting the Hydraulic Conductivity of Unsaturated Soils. Soil Sci. Soc. Am. J. 1980, 44, 892–898. 10.2136/sssaj1980.03615995004400050002x.

[ref40] Feddes R. A.; Kowalik P. J.; Zaradny H. Simulation of Field Water Use and Crop Yield*.*; PUDOC: Wageningen, 1978.

[ref41] Šimůnek J.; Hopmans J. W. Modeling Compensated Root Water and Nutrient Uptake. Ecol. Modell. 2009, 220, 505 10.1016/j.ecolmodel.2008.11.004.

[ref42] Durner W.; Jansen U.; Iden S. C. Effective Hydraulic Properties of Layered Soils at the Lysimeter Scale Determined by Inverse Modelling. Eur. J. Soil Sci. 2008, 59, 114–124. 10.1111/j.1365-2389.2007.00972.x.

[ref43] Feroz F.; Hobson M. P.; Bridges M. MultiNest: An Efficient and Robust Bayesian Inference Tool for Cosmology and Particle Physics. Mon. Not. R. Astron. Soc. 2009, 398, 1601–1614. 10.1111/j.1365-2966.2009.14548.x.

[ref44] Brunetti G.; Šimůnek J.; Glöckler D.; Stumpp C. Handling Model Complexity with Parsimony: Numerical Analysis of the Nitrogen Turnover in a Controlled Aquifer Model Setup. J. Hydrol. 2020, 584, 124681 10.1016/j.jhydrol.2020.124681.

[ref45] Brunetti G.; Papagrigoriou I.-A.; Stumpp C. Disentangling Model Complexity in Green Roof Hydrological Analysis: A Bayesian Perspective. Water Res. 2020, 182, 115973 10.1016/j.watres.2020.115973. 32673862

[ref46] Liu P.; Elshall A. S.; Ye M.; Beerli P.; Zeng X.; Lu D.; Tao Y. Evaluating Marginal Likelihood with Thermodynamic Integration Method and Comparison with Several Other Numerical Methods. Water Resour. Res. 2016, 52, 734–758. 10.1002/2014WR016718.

[ref47] Elsheikh A. H.; Wheeler M. F.; Hoteit I. Nested Sampling Algorithm for Subsurface Flow Model Selection, Uncertainty Quantification, and Nonlinear Calibration. Water Resour. Res. 2013, 49, 8383–8399. 10.1002/2012WR013406.

[ref48] Skilling J. Nested Sampling for General Bayesian Computation. Bayesian Anal. 2006, 1, 833 10.1214/06-BA127.

[ref49] Wesseling J.; Elbers J.; Kabat P.; Van den Broek B. SWAP 1993: Instructions for Input; Landbouwuniversiteit: *Internal Note, Winand Staring Centre*. Wageningen 1991.

[ref50] Tarantola S.; Gatelli D.; Mara T. A. Random Balance Designs for the Estimation of First Order Global Sensitivity Indices. Reliab. Eng. Syst. Saf. 2006, 91, 717–727. 10.1016/j.ress.2005.06.003.

[ref51] Tissot J. Y.; Prieur C. Bias Correction for the Estimation of Sensitivity Indices Based on Random Balance Designs. In Reliability Engineering and System Safety; Elsevier, 2012; Vol. 107, pp. 205–213. 10.1016/j.ress.2012.06.010.

[ref52] Saltelli A.; Tarantola S.; Chan K. P.-S. A Quantitative Model-Independent Method for Global Sensitivity Analysis of Model Output. Technometrics 1999, 41, 39–56. 10.2307/1270993.

[ref53] Satterthwaite F. E. Random Balance Experimentation. Technometrics 1959, 1, 111 10.1080/00401706.1959.10489853.

[ref54] Saltelli A.; Tarantola S. Sensitivity Analysis in Practice: A Guide to Assessing Scientific Models; Wiley: 2004, 232.

[ref55] Gelman A.; Carlin J. B.; Stern H. S.; Rubin D. B. Bayesian Data Analysis; Third Edition. CRC Press: 2004. 10.1186/1754-1611-9-2.

[ref56] Lu J.; Zhang Q.; Werner A. D.; Li Y.; Jiang S.; Tan Z. Root-Induced Changes of Soil Hydraulic Properties – A Review. J. Hydrol. 2020, 589, 125203. 10.1016/j.jhydrol.2020.125203.

[ref57] Gredelj A.; Polesel F.; Trapp S. Model-Based Analysis of the Uptake of Perfluoroalkyl Acids (PFAAs) from Soil into Plants. Chemosphere 2020, 244, 125534 10.1016/j.chemosphere.2019.125534. 32050335

[ref58] Li H.; Sheng G.; Chiou C. T.; Xu O. Relation of Organic Contaminant Equilibrium Sorption and Kinetic Uptake in Plants. Environ. Sci. Technol. 2005, 39, 4864 10.1021/es050424z. 16053085

[ref59] Dettenmaier E. M.; Doucette W. J.; Bugbee B. Chemical Hydrophobicity and Uptake by Plant Roots. Environ. Sci. Technol. 2009, 43, 324 10.1021/es801751x. 19238959

[ref60] Collins C.; Fryer M.; Grosso A. Plant Uptake of Non-Ionic Organic Chemicals. Environ. Sci. Technol. 2006, 40, 45 10.1021/es0508166. 16433331

[ref61] Zhang J.; Zhao W.; Pan J.; Qiu L.; Zhu Y. Tissue-Dependent Distribution and Accumulation of Chlorobenzenes by Vegetables in Urban Area. Environ. Int. 2005, 31, 855 10.1016/j.envint.2005.05.034. 16002141

[ref62] Legind C. N.; Rein A.; Serre J.; Brochier V.; Haudin C. S.; Cambier P.; Houot S.; Trapp S. Simultaneous Simulations of Uptake in Plants and Leaching to Groundwater of Cadmium and Lead for Arable Land Amended with Compost or Farmyard Manure. PLoS One 2012, 7, e47002 10.1371/journal.pone.0047002. 23056555 PMC3464289

[ref63] Honti M.; Hahn S.; Hennecke D.; Junker T.; Shrestha P.; Fenner K. Bridging across OECD 308 and 309 Data in Search of a Robust Biotransformation Indicator. Environ. Sci. Technol. 2016, 50, 6865 10.1021/acs.est.6b01097. 27213716

[ref64] Honti M.; Fenner K. Deriving Persistence Indicators from Regulatory Water-Sediment Studies - Opportunities and Limitations in OECD 308 Data. Environ. Sci. Technol. 2015, 49, 5879 10.1021/acs.est.5b00788. 25958980

[ref65] Brock A. L.; Rein A.; Polesel F.; Nowak K. M.; Kästner M.; Trapp S. Microbial Turnover of Glyphosate to Biomass: Utilization as Nutrient Source and Formation of AMPA and Biogenic NER in an OECD 308 Test. Environ. Sci. Technol. 2019, 53, 5838 10.1021/acs.est.9b01259. 30994338

[ref66] Ajami N. K.; Gu C. Complexity in Microbial Metabolic Processes in Soil Nitrogen Modeling: A Case for Model Averaging. Stoch. Environ. Res. Risk Assess. 2010, 24, 831 10.1007/s00477-010-0381-4.

[ref67] Mai T. H.; Schnepf A.; Vereecken H.; Vanderborght J. Continuum Multiscale Model of Root Water and Nutrient Uptake from Soil with Explicit Consideration of the 3D Root Architecture and the Rhizosphere Gradients. Plant Soil 2019, 439, 273 10.1007/s11104-018-3890-4.

[ref68] Delli Compagni R.; Gabrielli M.; Polesel F.; Turolla A.; Trapp S.; Vezzaro L.; Antonelli M. Risk Assessment of Contaminants of Emerging Concern in the Context of Wastewater Reuse for Irrigation: An Integrated Modelling Approach. Chemosphere 2020, 242, 125185 10.1016/j.chemosphere.2019.125185. 31689637

