# Supplementary material for: On the Use of Mechanistic Soil–Plant Uptake Models: A Comprehensive Experimental and Numerical Analysis on the Translocation of Carbamazepine in Green Pea Plants
Source: Environ Sci Technol. 2021 Feb 15;55(5):2991–3000. doi: 10.1021/acs.est.0c07420 (PMC8023655; doi:10.1021/acs.est.0c07420)
Supplement: Supplementary file 1 — es0c07420_si_001.pdf [file es0c07420_si_001.pdf]

Supplementing Information

ON THE USE OF MECHANISTIC SOIL-PLANT UPTAKE MODELS: A COMPREHENSIVE EXPERIMENTAL AND NUMERICAL ANALYSIS ON THE TRANSLOCATION OF CARBAMAZEPINE IN GREEN PEA PLANTS

Giuseppe Brunetti[a]*, Radka Kodešová[b], Helena Švecová[c], Miroslav Fér[b], Antonín Nikodem[b], Aleš Klement[b], Roman Grabic[c], Jiří Šimůnek[d]

[a] *University of Natural Resources and Life Sciences, Vienna (BOKU), Institute for Soil Physics and Rural Water Management, Muthgasse 18, 1180, Vienna, Austria*
[b] *Czech University of Life Sciences Prague, Faculty of Agrobiology, Food and Natural Resources, Dept. of Soil Science and Soil Protection, Kamýcká 129, CZ-16500 Prague 6, Czech Republic*
[c] *University of South Bohemia in České Budějovice, Faculty of Fisheries and Protection of Waters, South Bohemian Research Center of Aquaculture and Biodiversity of Hydrocenoses, Zátiší 728/II, CZ-38925 Vodňany, Czech Republic*
[d] *University of California, Riverside, Department of Environmental Sciences, CA 92521, USA*

*Corresponding Author: giuseppe.brunetti@boku.ac.at

Number of Pages: 14

Number of Tables: 7

Number of Figures: 7

Analytical methods

Chemicals

Analytical standards of native compounds and isotope-labeled internal standards were purchased via Labicom (Olomouc, Czech Republic). The exact origin of individual standards is reported in our previous studies [36,37]. All standards were of analytical grade or high purity (>98%). A stock solution of each pharmaceutical was prepared in methanol at a concentration of 1 mg/mL. Analytical working solutions of individual compounds and their mixture were prepared by diluting (and mixing) the stock solutions with methanol to a final concentration of 1 µg/mL. All stock and spiking solutions were stored at -20 °C. The LC-MS grade acetonitrile and 2-propanol (LiChrosolv Hypergrade) were obtained from Merck (Darmstadt, Germany). Formic acid used for acidification of mobile phases was purchased from Labicom (Olomouc, Czech Republic) and was LC-MS grade. The ultrapure water was prepared using Aqua-MAX-Ultra System (Younglin, Kyounggi-do, South Korea).

Sample extraction

An ultrasound-based extraction approach with two solvent mixtures was applied to analyze CBZ and its metabolites in soil samples [36]. Briefly, about 2 g of each freeze-dried soil sample was placed into a 10-mL autosampler vial, and 20 ng of internal standard was added. The samples were then extracted with 4 mL of extraction mixture 1 (acetonitrile/water 1/1, v/v acidified with 0.1% of formic acid) followed with 4 mL of mixture 2 (acetonitrile/2-propanol/H_2O, 3/3/4, v/v/v, acidified with 0.1% of formic acid) in an ultrasonic bath (DT 255, Bandelin Electronic, Sonorex Digitec, Berlin, Germany) for 15 min. The supernatants were combined and filtered through a syringe filter (0.45 µm, regenerated cellulose, Labicom, Olomouc, Czech Republic). The freeze-dried plant samples were extracted as follows [16]: 0.05 g of sample was placed in an Eppendorf tube with a safe lock, 5 ng of internal standard, and stainless steel ball, and 1 mL of extraction mixture was added. Samples were consequently

extracted by shaking at 1 800 min^{-1} for 5 min (TissueLyser II, Quiagen, Germany). The samples were then centrifuged at 10 000 min^{-1} for 5 min (Mini spin centrifuge, Eppendorf), and the supernatant was filtered through a syringe filter (0.45 µm regenerated cellulose filters) to clean the Eppendorf tube. Aliquots of 100 µl were pipetted into an insert in an autosampler vial for LC-MS analysis.

Instrumental analysis

The concentrations of pharmaceuticals and their metabolites in supernatants from plant tissues and soils were determined using liquid chromatography-tandem mass spectrometry (LC–MS) and either isotope dilution or an internal standard (IS) method with using matrix matching standard. A triple-stage quadrupole mass spectrometer, Quantiva (Thermo Fisher Scientific, San Jose, CA, USA), coupled with an Accela 1250 LC pump (Thermo Fisher Scientific) and HTS XT-CTC autosampler (CTC Analytics AG, Zwingen, Switzerland), were used for the analysis of irrigation water [21]. A hybrid quadrupole - orbital trap mass spectrometer, Q Exactive™ HF Hybrid Quadrupole-Orbitrap™ Mass Spectrometer (Thermo Fisher Scientific, USA), coupled with a Vanquish Pumps (Dionex, Germany) and a PAL RSI autosampler (CTC Analytics AG, Switzerland) was used for the analysis of plant and soil samples. A Hypersil Gold aQ column (50 mm × 2.1 mm i.d., 5µm particle size, from Thermo Fisher Scientific San Jose, CA, USA) was used for the chromatographic separation of the target compounds. A detailed description of the instrument settings can be found in Grabicova et al. [37]. More information about conditions of analysis, including gradient elution conditions, m/z values, and retention time, is provided in Table S1. The matrix effects were corrected using a matrix matching standard.

Method validation

The above described analytical method for soil analyses was validated for a wide range of compounds (including CBZ and its metabolites) and a wide range of soils (including the soil, which is used in this study) [36,37]. The method for the analysis of plant tissues was validated for CBZ and its metabolites for three leaf vegetables (arugula, spinach, and lamb's lettuce) and radish plants [16,17] and for green pea plants [23]. Despite that, the method was initially tested at a fortification level of 100 ng g^{-1} and 1000 ng g^{-1} for soils and plants, respectively (Table S4). No corrections were assumed with respect to recoveries when analyzing concentrations in soil and plant tissues. In addition, duplicates of every third sample were analyzed. The limits of quantification (LOQ) of particular compounds in all matrices are presented in Table S5.

Table S1. Basic soil characteristics of the Haplic Chernozem on loess: pH_{H2O}, pH_{KCl}, pH_{CaCl2}, organic carbon content (Cox), salinity, cation exchange capacity (CEC), soil hydrolytic acidity (HA), basic cation saturation (BCS), sorption complex saturation (SCS), soil particle density (ϱ_s), clay, silt and sand contents, nitrogen (N), phosphorus (P) and potassium (K) contents.

pH_{H2O}	pH_{KCl}	pH_{CaCl2}	Cox (%)	Salinity (μS cm^{-1})	Clay (%)	Silt (%)	Sand (%)
8.08	7.04	7.35	1.75	97.2	36.5	58.1	5.4
ϱ_s (g cm^{-3})	CEC ($mmol^+kg^{-1}$)	HA ($mmol^+kg^{-1}$)	BCS ($mmol^+kg^{-1}$)	SCS (%)	N (mg kg^{-1})	P (mg kg^{-1})	K (mg kg^{-1})
2.53	235.0	4.21	230.8	98.2	18.6	135	340

Table S2. Irrigation doses and carbamazepine concentrations.

Day	Irrigation dose (cm^3)	Concentrations of carbamazepine (ng cm^{-3})
0	50	0
1	75	0
3	50	0
6	100	0
7	100	0
9	100	0
10	150	0
13	150	0
14	100	0
16	150	120
17	100	120
20	100	99
21	100	99
23	100	99
24	200	89
27	200	89
28	100	89
30	200	99
31	200	99
32	200	99
35	250	110
37	200	110

38	200	110
41	250	100
42	100	100
43	300	100
44	100	100
45	300	100

Table S3. Information about LC-MS/MS parameters: A, B are mobile phases, A - H2O (with 0.1 % formic acid, FA), B - acetonitrile (with 0.1 % FA).

Gradient elution conditions				
Time [min]		A [%]	B [%]	Flow [µL min^{-1}]
0		100	0	350
1		100	0	350
4		75	25	350
8		40	60	450
10		0	100	450
11.5		0	100	450
11.55		100	0	350
13		100	0	350
Mass spectrometry conditions				
Compound	Parent ion	m/z quan	m/z qual	Retention time [min]
Carbamazepine	237.1022	194.0964	192.0808	5.96
Carbamazepine 10,11-epoxide	253.0972	210.0913	180.0811	5.30
trans -10,11- dihydro 10,11- dihydroxy carbamazepine	271.1177	254.0813	210.0915	4.66
10,11-dihydrocarbamazepine	239.1179	194.0965	222.0913	6.02
Oxcarbazepine	253.0972	236.0707	208.0757	5.48

Table S4. Compounds recovery 200 ng per 2 g of soil and 50 ng per 0.05 g of plant tissues (%).

Compound	Soil	Roots	Stem	Leaves	Fruits
Carbamazepine	135%	84%	95%	99%	94%
Carbamazepine 10,11-epoxide	104%	114%	111%	130%	108%
trans -10,11- dihydro 10,11- dihydroxy carbamazepine	155%	81%	111%	111%	108%

10,11-dihydrocarbamazepine	73%	83%	121%	111%	98%
Oxcarbazepine	85%	85%	91%	121%	96%

Table S5. Limits of compounds' quantification (ng g^{-1}).

Compound	Soil		Roots		Stem		Leaves		Fruits	
	min	max	min	max	min	max	min	max	min	max
Carbamazepine	0.27	2.9	0.074	0.25	0.074	0.21	0.074	0.21	0.074	0.26
Carbamazepine 10,11-epoxide	0.3	3.2	0.022	0.075	0.022	0.063	0.022	0.086	0.022	0.1
10,11-dihydrocarbamazepine	0.24	2.5	0.081	0.27	0.081	0.24	0.081	0.24	0.081	0.28
trans -10,11- dihydro 10,11-dihydroxy carbamazepine	0.14	1	0.48	1.1	0.55	1.6	0.55	2.2	0.55	1.5
Oxcarbazepine	1.3	13	0.025	0.087	0.025	0.072	0.025	0.098	0.025	0.088

Table S6. Concentrations of carbamazepine and carbamazepine 10,11-epoxide in plant tissues (ng g^{-1} dry weight). Concentrations of other metabolites were mostly below the limits of quantification (LOQ).

Carbamazepine	Roots		Stem		Leaves		Fruits	
Sampling day	Avg.	St. Dev.	Avg.	St .Dev.	Avg.	St. Dev	Avg.	St. Dev.
23	60.7	24.8	20.1	4.7	41.0	7.3	NA	
30	113.4	18.1	83.8	12.2	49.4	11.0	NA	
41	180.4	28.6	102.0	4.7	45.3	3.0	29.6	7.2
48	317.2	71.1	148.8	28.8	75.1	13.3	44.8	2.8
Carbamazepine 10,11-epoxide	Roots		Stem		Leaves			
Sampling day	Avg.	St. Dev.	Avg.	St.Dev.	Avg.	St. Dev.	Avg.	St.Dev.
23	2.1	0.5	2.1	0.7	128.8	16.7	NA	
30	3.2	0.6	4.9	1.6	293.8	87.3	NA	
41	7.3	1.3	14.8	5.9	490.0	46.4	3.6	1.1
48	11.9	2.3	29.5	7.2	892.5	45.5	11.6	4.1

Table S7. Concentrations of carbamazepine and carbamazepine 10,11-epoxide in soils (ng g^{-1} dry weight). Concentrations of metabolites were mostly below the limits of quantification (LOQ).

Carbamazepine	0-5 cm		5-10 cm		10-15 cm		15-20 cm	
Sampling day	Avg.	St.Dev.	Avg.	St.Dev.	Avg.	St.Dev.	Avg.	St.Dev.
23	28.4	13.9	2.4	1.6	1.2	0.5	2.0	1.2

30	112.7	75.4	9.1	4.2	4.9	4.2	11.2	6.0
41	191.3	37.0	10.5	3.3	10.8	4.2	4.3	1.8
48	222.0	130.9	26.1	14.7	9.5	7.1	3.9	1.9
Carbamazepine 10,11-epoxide	0-5 cm		5-10 cm		10-15 cm		15-20 cm	
Sampling day	Avg.	St.Dev.	Avg.	St.Dev.	Avg.	St.Dev.	Avg.	St.Dev.
41	3.63	1.46	<LOQ		<LOQ		<LOQ	
48	3.89	2.76	<LOQ		<LOQ		<LOQ	

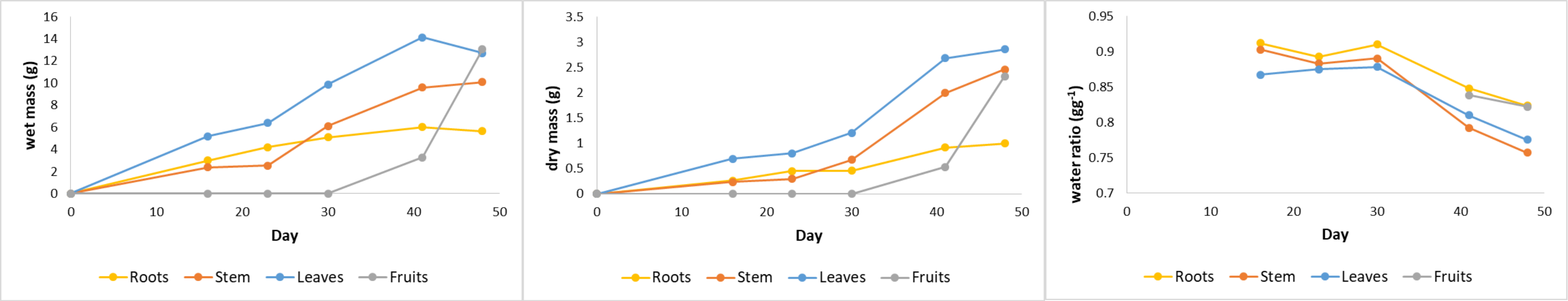

Figure S1. The total wet and dry masses and water ratios of plant tissues in each column. A weighted average of root masses at different soil depths is used for the root compartment.

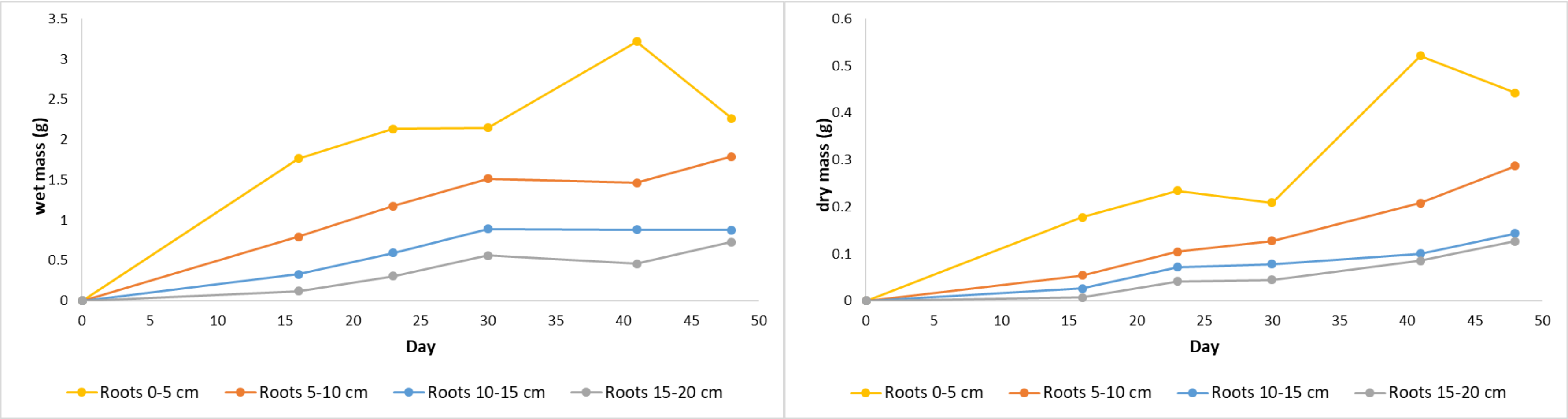

Figure S2. The wet and dry masses of roots at different soil depths.

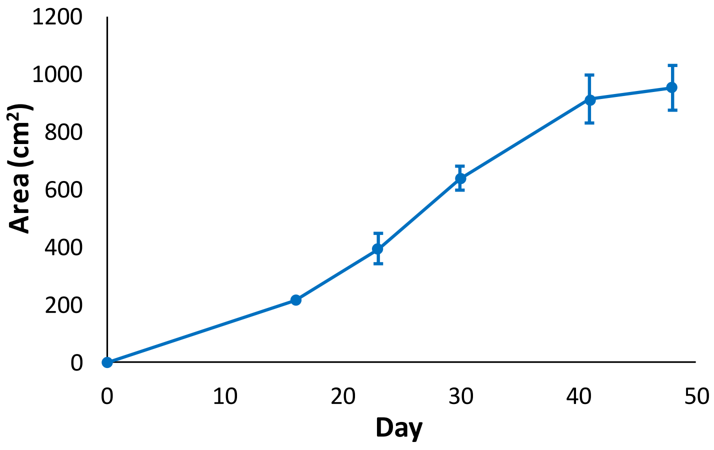

Figure S3. The total area of leaves in each column evaluated on scanned fresh leaves using the ImageJ software.

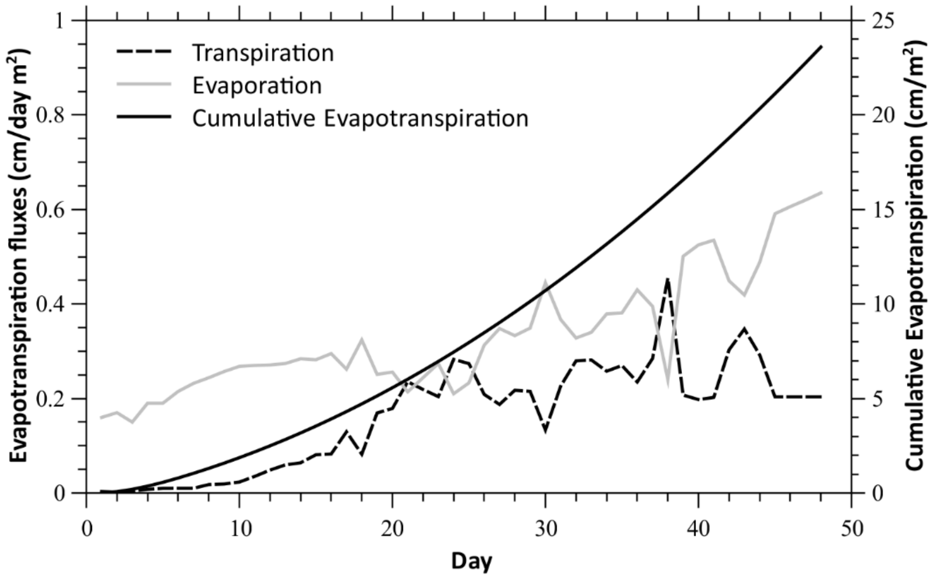

Figure S4. Measured transpiration (dashed), evaporation (grey), and cumulative evapotranspiration (solid) during the experiment.

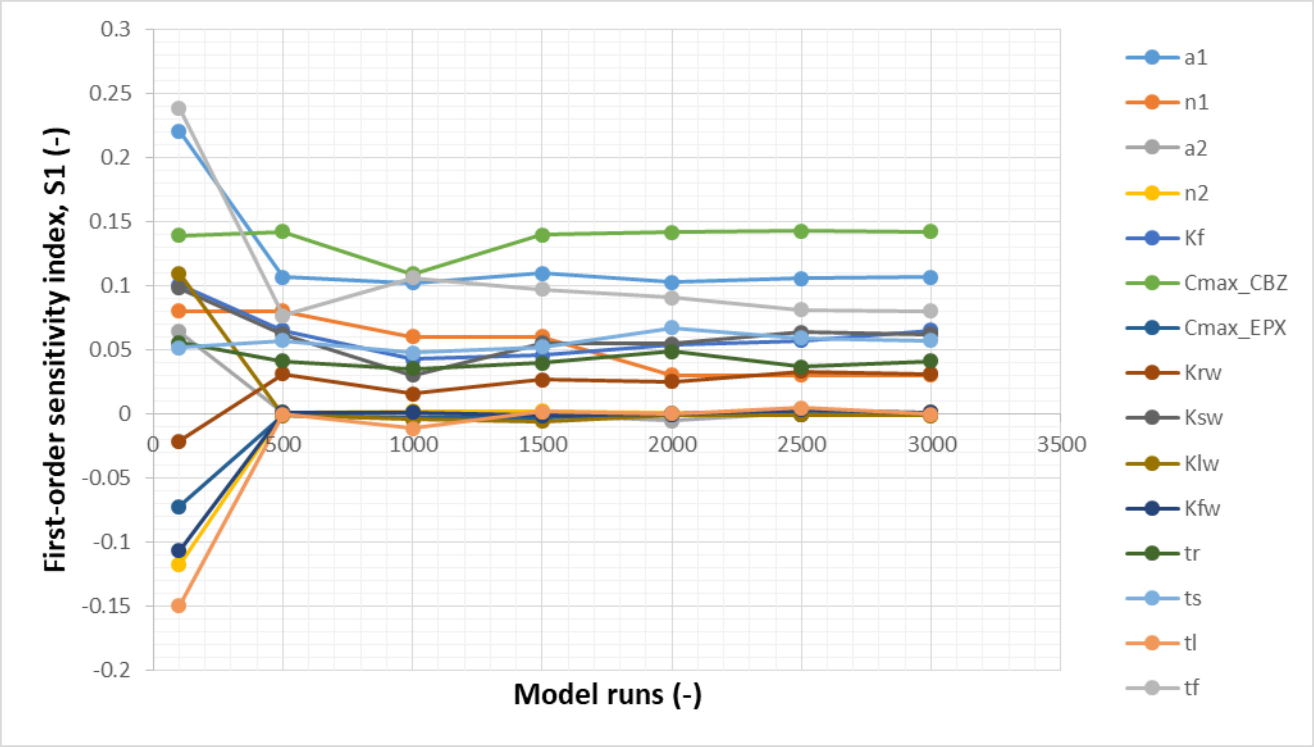

Figure S5. Convergence analysis of first-order sensitivity indices.

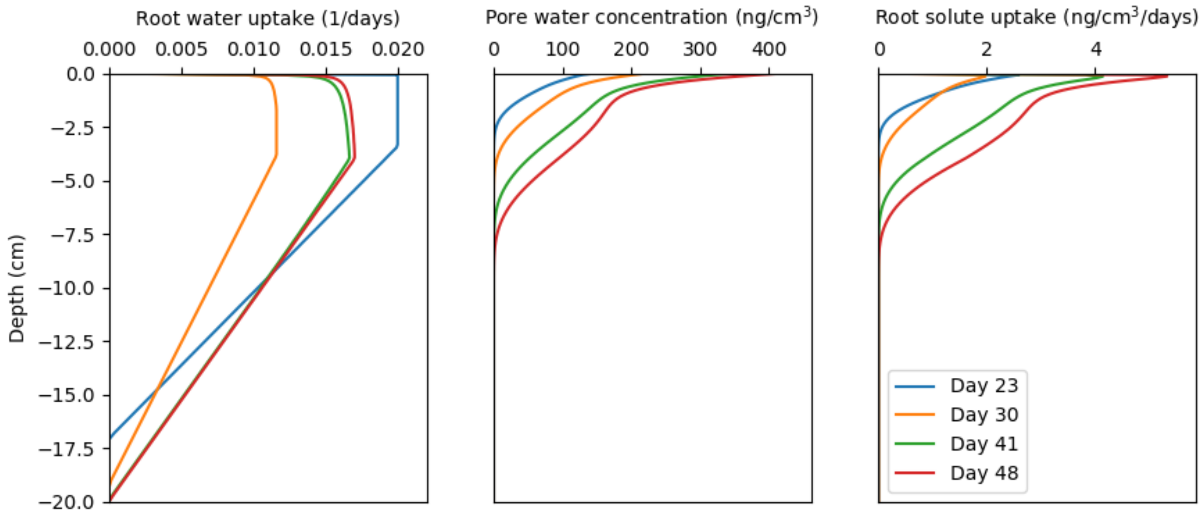

Figure S6. Simulated root water uptake, pore water concentration, and root solute uptake along the soil profile at different times.

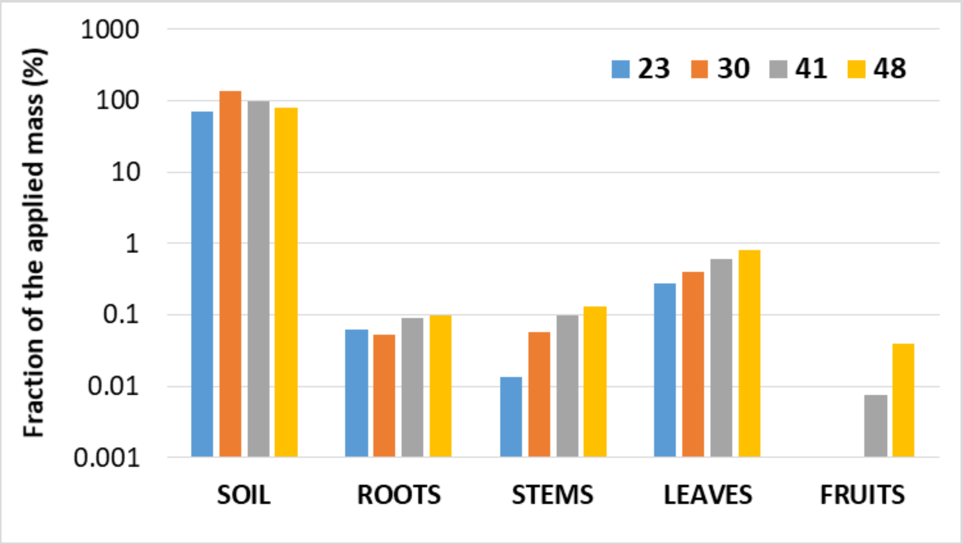

Figure S7. Measured average fractions of the applied solute mass (the sum of CBZ and EPX) in the soil and different plant compartments at different times.
